# Genotype-Independent Transformation and Genome Editing of *Brassica napus* Using a Novel Explant Material

**DOI:** 10.3389/fpls.2020.579524

**Published:** 2020-10-08

**Authors:** Uyen Cao Chu, Sandeep Kumar, Amy Sigmund, Kari Johnson, Yinghong Li, Pamila Vongdeuane, Todd J. Jones

**Affiliations:** Corteva Agriscience, Johnston, IA, United States

**Keywords:** canola, internode, genotype independent, transformation, gene editing

## Abstract

*Agrobacterium*-mediated transformation of canola (*Brassica napus*) via hypocotyl segments has been a commonly used method for the past 30 years. While the hypocotyl-based method is well-established, it is not readily adapted to elite germplasm and the prolonged process is not ideal for a production transformation setting. We developed an *Agrobacterium*-mediated transformation method based on epicotyl and higher stem (internodal) segments that is efficient, rapid and amenable for high-throughput transformation and genome editing. The method has been successfully implemented in multiple canola genotypes. The method appears to be genotype-independent, with varying transformation efficiencies. Internodal segment transformation was used to generate transgenic events as well as CRISPR-Cas9-mediated frameshift gene knockouts.

## Introduction

Canola, and the related rapeseed, *Brassica napus* L. subsp. *napus*, is the second most important oilseed crop in the world, ranking only behind soybean in production and value (Foreign Agricultural Service/USDA, 2020). Canola is an allotetraploid species with two genomes derived from a wide cross between *B. rapa* (2n = 20, the “A” genome) and *Brassica oleracea* (2n = 18, the “C” genome). Genetic modification of canola has been possible for many years, with the first report in 1987 (Fry et al., 1987). However, transformation and plant regeneration of canola has long been recognized as being genotype-dependent (Poulsen, 1996; Boszoradova et al., 2011) *Agrobacterium*-mediated transformation of canola via hypocotyl segments has been a commonly used method for the past 30 years (Radke et al., 1988; De Block et al., 1989; Cardoza and Stewart, 2003; Maheshwari et al., 2011). The hypocotyl system has worked well for a limited range of genotypes, with Westar being the most commonly employed genotype (De Block et al., 1989; Cardoza and Stewart, 2003). Other researchers have been able to extend hypocotyl transformation to a few other genotypes using a modified version of the protocol with no selection (Maheshwari et al., 2011), it has been challenging to make the hypocotyl system work efficiently for elite commercial canola germplasm representing a broad range of breeding genetics (Hao et al., 2010; Farooq et al., 2019).

Boszoradova et al. (2011) modified the hypocotyl-based method by lowering the selection pressure and delaying the application of selection by 14 days. Consequently, they were able to transform three “economically important” spring rapeseed varieties with efficiencies ranging from 1.0 to 5.5%, but they were unsuccessful with a fourth cultivar (Campino). Zhang et al. (2005) successfully transformed four Australian wheat cultivars using 4 day-old seedling-grown cotyledon or hypocotyls explants. Transformation efficiencies from hypocotyl-based experiments ranged from 5.3 to 32.8%, demonstrating that some commercial Australian germplasm can be transformed with relatively high efficiency. Recently, Braatz et al. (2017) demonstrated CRISPR-Cas targeted mutagenesis in canola cv “Haydn” using a hypocotyl-based method similar to that described by De Block et al. (1989). They regenerated four shoots from 625 hypocotyl explants after 9–11 months in tissue culture.

Other transformation systems for canola have been reported previously, including methods based on microspores as the explant and particle bombardment or *Agrobacterium* transformation as the delivery method (Huang, 1992; Fukuoka et al., 1998; Chen and Tulsieram, 2015). Transformation of embryos derived from microspores and infected with *Agrobacterium* has also been described (Cegielska-Taras et al., 2008). While transforming haploid microspores or microspore-derived embryos is attractive because the process produces homozygous double-haploid plants, however the process is lengthy, laborious and genotype-dependent (Cegielska-Taras et al., 2008; Chen and Tulsieram, 2015). Leaf tissue, cotyledons, and petioles have also been described in the literature as having regeneration potential (Akasaka-Kennedy et al., 2005; Farooq et al., 2019) but these explant types have not been widely adopted for canola transformation. For a comprehensive review of *Brassica* transformation methodology, please see Rani et al. (2013). Hybrid canola has come to dominate the commercial canola market. Being able to transform or edit the male and female parents of current hybrids is imperative for the development of new traits to be deployed as hybrids. Our goal was to develop an efficient and robust transformation method that could be used with a broad range of Corteva canola germplasm, representing both male and female heterotic groups. To the best of our knowledge, there are no reports of transformation of elite canola parental lines that are used in the production of commercial canola hybrids.

In this report, we describe a transformation method for canola that is simple, rapid, robust, and applicable for a broad range of commercial, elite canola germplasm, from both male and female heterotic groups. The method utilized canola seedling internodal segments (epicotyl and stem segments) and relied on the *spcN* gene and spectinomycin selection for efficient selection of transgenic events. This protocol was successfully tested in a range of elite canola lines, representing both male and female genotypes, and consistently produced events with few to no escapes. T0 plants exhibited a normal phenotype, were fertile and transmitted the transgene to the next generation. The protocol was also used to generate CRISPR-Cas-mediated edited events with mutations detected in both the A and the C genomes.

## Materials and Methods

### Donor Material Preparation

Twelve elite canola genotypes were utilized in this experiment. Seed was obtained from a Corteva seed storage facility, located in Georgetown, ON, Canada.

**FIGURE 1:**
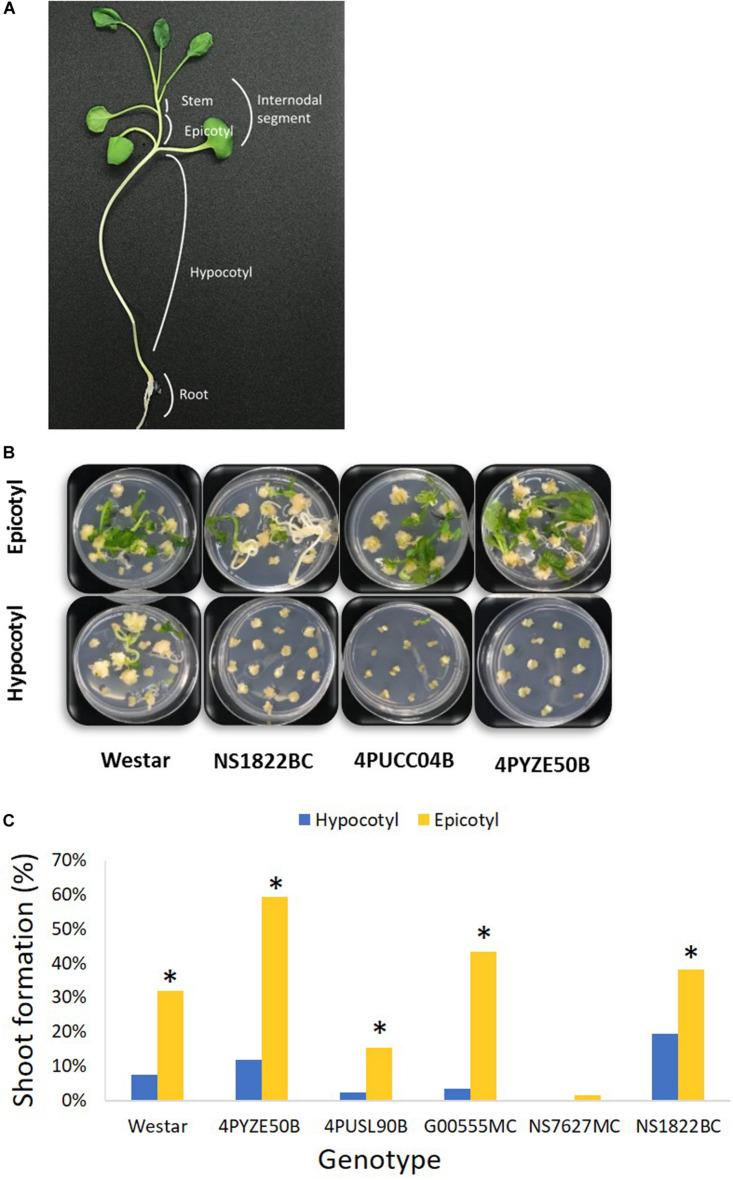
Hypocotyl and epicotyl explants from multiple genotypes. **(A)** A diagram of canola seedling; **(B)** representative shoot formation from epicotyl versus hypocotyl explants for multiple genotypes at day 42 after infection; **(C)** percentage shoot formation from epicotyl and hypocotyl explants at day 42 after infection. Epicotyl explants were significant better than hypocotyl in shoot formation in every genotype. Genotype NS7627MC had no response from hypocotyl. *P < 0.05 [Student’s *t*-test, JMP 14 software (SAS institute, Cary, NC)].

Prior to transformation, canola seeds were germinated aseptically to produce seedlings for explant material. Canola seeds were soaked in 70% ethanol and agitated using a stir plate for 2 min, and further surface-sterilized for 10 min with a 20% bleach solution made with diH_2_O and Clorox bleach (6.15% sodium hypochlorite) with the addition of 2–4 drops of Tween 20. Seeds were then rinsed with copious amounts of sterile diH_2_O. Canola seeds were germinated in Stericon^TM^ vessels (PhytoTechnology Labs, Shawnee Mission, KS) on half-strength MS medium, 90 base (Supplementary Table 1), at 26°C in a culture room with cool white fluorescent lights (GE, United States), 16 h daylength, 45 μmol/m^2^/s, for 18–29 days, to obtain an epicotyl of from 1 to 4 cm in length (Figure 1A). Twelve to fourteen seeds were germinated in one Stericon^TM^ vessel or similar container. For the comparison of the size of explant, seedlings of the female genotype 4PYZE50B grown in Stericon^TM^ vessels were used. To evaluate the impact of lighting conditions on explant responsiveness, canola seeds of genotype 4PYZE50B were germinated and grown in Stericon^TM^ vessels for 7–10 days as above, after which germinated seedlings with 1–2 cm of visible epicotyl were transferred to either LED lights, 16 h daylength, at 60 μmol/m^2^/s (BX Series, Valoya, Finland), or cool white fluorescent lights, 16 h daylength, at 45 μmol/m^2^/s (GE, United States) or dark, all at 26°C (Table 4). Explants, 3–4 mm segments of epicotyl and higher stem segments, specifically excluding any tissue from the nodes collectively and called “internodal segments,” were collected at 11–18 days after transfer to LED lighting.

### Plasmid Construction and Creation of *Agrobacterium tumefaciens* Strain for Plant Transformation

**FIGURE 2:**
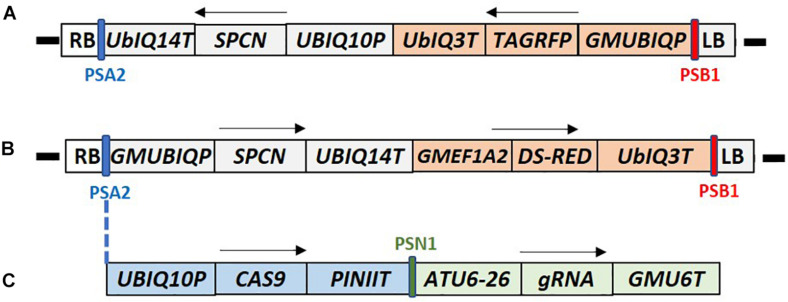
T-DNA maps of the constructs used for canola transformation. **(A)** Construct RV008033 T-DNA contains Arabidopsis UBIQ10 promoter driven *SPCN* selectable marker, and *TAG-RFP* fluorescent marker gene under the control of the soybean UBQ promoter (GMUBIQP); **(B)** Construct RV028667 T-DNA contains the soybean UBQ promoter driven *SPCN* selectable marker, and *Ds-RED* fluorescent marker gene driven by the soybean elongation factor (GMEF1A2) promoter; **(C)** Construct RV29164 T-DNA contains *SPCN* selectable marker, and *Ds-RED* fluorescent marker gene similar to RV028667. In addition it also contains single guide RNA (sgRNA) and CAS9 expression cassette inserted between PSA2 and GMUBIQP. T-DNA components shown in these maps are described in Supplementary Table 2. PSA2, PSN1, SPCN, and PSB2 regions of the maps were used for Q-PCR detection as described in Supplementary Table 3. RB and LB indicate T-DNA right and left border, respectively. Arrows indicate direction of the transcription. Sizes are not to the scale.

Standard molecular methods and Gateway^®^ (Thermo Fisher Scientific) cloning were used to construct the plasmids used in this report. The DNA components of different plasmids used in this study are described in the Supplementary Table 2. Plasmids RV008033 and RV028667 were used for optimization of plant transformation (Figures 2A,B). The T-DNA of both plasmids contains different fluorescent marker genes, and the *spcN* gene, conferring resistance to spectinomycin, as the plant selectable marker driven by different promoters as shown in Figure 2. Plasmid RV029164 (Figure 2C) was created to test CRISPR-Cas9-mediated targeted mutagenesis in canola. In addition to the spectinomycin selectable marker (*spcN*) and DsRed fluorescent gene, the plasmids contained a Cas9 expression cassette, and a single guide RNA (sgRNA) consisting of the Arabidopsis U6 polymerase III promoter, a CRISPR RNA, a trans-activating CRISPR RNA and a soybean U6 terminator as described in Supplementary Table 2. The DNA sequence of the canola *Caffeoyl-CoA O-methyltransferase* (*COMT*) gene (Boerjan et al., 2003) was scanned using Cas-OFFinder (Bae et al., 2014) for unique sequence regions and potential target sites were identified by first locating a suitable PAM recognition sequence for *Streptococcus pyogenes* Cas9, NGG and then extracting ∼20 bp of sequence 5′ of the PAM for use as the spacer (gagaagatcaaggtctagcg) in the sgRNA. Potential off-target sites for the selected genomic target were evaluated by searching the public *B. napus* genome.

### Explant Preparation and Transformation

Transformation vectors were moved into the auxotrophic *Agrobacterium tumefaciens* strain LBA4404 Thy- (Ranch et al., 2012) containing the helper plasmid pVir9 (Anand et al., 2018). *Agrobacterium* was suspended in 20A medium (Supplementary Table 1) and adjusted to an O.D. of 1.0 at 600 nm (Genesys 30, Thermo Fisher Scientific, Waltham, MA). The base protocol for tissue culture and transformation conditions was derived from De Block et al. (1989). Epicotyl and higher stem internodes were sliced into 3–4 mm segments (internodal segments) in a 100 × 25 mm petri dish containing 12 ml of 20A medium, 24 μl of *Agrobacterium* suspension supplemented with 12 μl of 100 mM acetosyringone. The *Agrobacterium* suspension including explants was gently shaken at 56 rpm on a platform shaker (VWR Advanced Digital Shaker Model 15000, VWR Scientific, Radnor, PA) for 10 min and then co-cultivated under low light (12 μmol/m^2^/s) at 21°C for 2–4 days (optimal 3 days) in a growth chamber (Percival Scientific model #LED41HL2, Perry, IA). The internodal segments were transferred onto shoot formation medium 70A which contains 5 mg/L spectinomycin dihydrochloride and 0.1 g/L myo-inositol, 0.5 mg/L nicotinic acid, 0.1 mg/L thiamine HCl, 0.5 mg/L pyridoxine HCl, 2 mg/L glycine, 0.5 g/L MES buffer, 0.04 g/L adenine hemisulfate salt, 20 g/L sucrose, 0.5 g/L PVP-40, 0.1 mg/L NAA, 1 mg/L BAP, 0.01 mg/L gibberellic acid, 2 mg/L silver nitrate, 500 mg/L carbenicillin (Supplementary Table 1) for 2 weeks and sub-cultured for an additional 2–4 weeks or until small shoots developed at 26°C. Small shoots were transferred to 70C medium supplemented with 10 mg/L spectinomycin dihydrochloride, 0.0025 mg/L BAP and, 20 g/L sucrose (Supplementary Table 1) for shoot elongation for 2 weeks or until the shoot was 1 cm long. Shoots were transferred onto 90A medium containing 10 g/L sucrose, 0.5 mg/L indole 3-butyric acid with no selection (Supplementary Table 1) for rooting. All tissue culture steps were maintained at 26°C. When roots formed, the putative T0 plantlets were sent to the greenhouse and transferred into Elle plugs (Ellepot AS, Esbjerg, Denmark) with 200 mix substrate (peat, perlite, vermiculite, lime, starter, and wetting agent) and placed into 8 × 4 flats. Putative T0 plantlets are defined as spectinomycin-resistant plantlets prior to pPCR molecular confirmation.

Equations for percentage calculations are as follows: % shoot = (#shoots/#explants) × 100; % T0 plant = (#T0 plants/#explants) × 100; % PCR+ = (#PCR+/#T0 plants) × 100; % quality event = (#Single Copy, no vector backbone events/#T0 plants) × 100. Statistical analysis was done using JMP 14 software (SAS institute, Cary, NC).

The optimized transformation protocol utilized seedlings grown for 11–18 days under LED light (60 μmol/m^2^/s) after growing under fluorescent lighting (45 μmol/m^2^/s) for 7–10 days (Supplementary Figure 2). Total maximum seedling age is 21–28 days. The optimal internodal segment size is 3–4 mm. *spcN* gene and spectinomycin selection (5 mg/L at shoot formation and 10 mg/L at shoot elongation) were used for transformation. All tissue culture steps were performed at 26°C.

### Plant Molecular Analysis

After transfer to flats, plants were sampled by leaf punching at 7 days after transplantation for quantitative PCR (qPCR) to confirm the presence of the selectable marker (*spcN*), CRISPR-Cas9 (PSN1), the left (PSB1) and right border (PSA2) regions, and the absence of *A. tumefaciens* backbone sequences (Ingham et al., 2001) following the protocol of Wu et al. (2014). The *spcN*, PSN1, PSB1, and PSA2 regions for qPCR are shown in T-DNA maps in Figure 2. The primers and probes used for qPCR assays are described in Supplementary Table 3. DNA was extracted from the 7 mm leaf disk with sbeadex^TM^ chemistry (LGC Biosearch, Middlesex, United Kingdom) as per vendor recommendations and resulting DNA was analyzed for quality and quantity via DropSense (Unchained Labs, Pleasanton, CA). To identify correctly edited targets, variants were analyzed via two-step PCR amplification and Illumina sequencing. In brief, target site amplification primers were designed via Primer3 (Untergasser et al., 2012), such that resulting amplification products were 75–250 bp in length and contained the target cut site. Overhang sequences were added to the target site primers to facilitate addition of sample index identifiers and Illumina specific sequences. Primary PCR amplifications were performed using 10 μl 2× Phusion Flash master mix (Thermo Fisher Scientific, Waltham, MA). Secondary PCR amplifications were performed to incorporate sample specific indices and Illumina specific sequences with 5 μl of treated primary PCR product. Amplicon pools were purified with AmpureXP (Beckman Coulter, Pasadena, CA) purification beads, 1:1 amplicon:beads as per vendor specifications. Purified pools were checked for quality and quantity with the Agilent Bioanalyzer DNA 7500 kit, normalized to 2 nM, denatured according to Illumina sequencing protocols, hybridized, clustered, and sequenced on a MiSeq (Illumina, San Diego, CA). Individual sample sequences were trimmed for adapter sequence with Cutadapt, version 1.9.1, aligned with BWA-MEM, version 0.7.13-r1126, and characterized for allele changes with SAM tools, version 1.3. Reads with a ≥1 nucleotide indel arising within a window centered over the expected cleavage site and not observed in the negative control were classified as NHEJ mutations. The total numbers of NHEJ mutations were then used to calculate the % mutant reads based on the total number of reads of an appropriate length containing a perfect match to the barcode and forward primer. Positive transgene- or edit-confirmed plants were transplanted to 200 mix substrate after 21 days, fertilized with 17-4-17 (NPK) 100 ppm, and grown at 68°F under 16 h daylength, at 350 μmol/m^2^/s in a growth chamber (Conviron BDW 120, Winnipeg, Manitoba).

## Results

### Selection Using the *spcN* Gene and Spectinomycin Selection for Canola Transformation

An initial test was done to evaluate the appropriate level of spectinomycin for effective selection using genotype NS1822BC (Table 1). Internodal segments were cut and transferred onto medium 70A containing either 5, 25, 50, and 100 mg/L spectinomycin and were subcultured to fresh media every 3 weeks. At 7 days, the explants remained green and healthy at all levels of spectinomycin. At 18 days, only the explants on 5 mg/L were visibly green and viable; explants on all other concentrations were bleached or dead. Based on this, we chose to use 5 mg/L spectinomycin for 6 weeks of selection during shoot initiation, followed by another 2 weeks on 10 mg/L for shoot elongation. No selection was used at the rooting stage.

**TABLE 1:** Spectinomycin dose response using NS1822BC.

		Spectinomycin concentration											
Rep	Explant#	5 mg/L			25 mg/L			50 mg/L			100 mg/L		
		7 days	18 days	34 days	7 days	18 days	34 days	7 days	18 days	34 days	7 days	18 days	34 days
Rep 1	12	+++	++	+	+++	Dead	N/A	+++	Dead	N/A	+++	Dead	N/A
Rep 2	12	+++	++	+	+++	Dead	N/A	+++	Dead	N/A	+++	Dead	N/A

*+++, indicates all explants responding, green tissue; ++, indicates moderate growth, some yellow tissue; +, indicates poor/no growth, bleached/necrotic tissue.*

**FIGURE 3:**
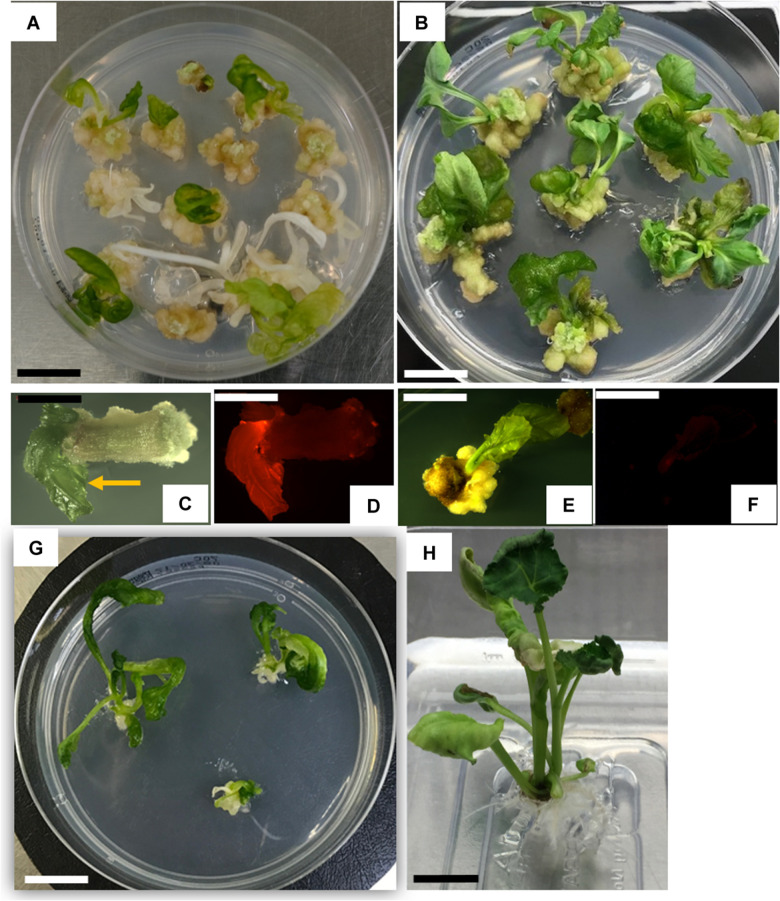
Canola transformation. **(A)** Transgenic (green) and non-transgenic (bleached) shoots at 31 days after infection; **(B)** wild-type internodal tissue culture with no selection at 31 days **(C,D)** transgenic shoot, brightfield **(C)**, and corresponding shoot expressing TagRFP **(D)**; **(E,F**) wild-type shoot, brightfield **(E)** and corresponding auto-fluorescent view **(F)**; **(G)** elongating shoots at 42 days after infection; **(H)** transgenic rooted plant. Scale bar for **A,B,G,H** = 1.0 cm; scale bar for **C–F** = 0.4 cm.

To establish an initial transformation protocol, 3–4 mm internodal segments were transferred to 100 × 25 mm petri plates and infected with LBA4404 Thy- *Agrobacterium* suspension containing the cassette RV008033 with the *spcN* gene for spectinomycin selection and TagRFP (Merzlyak et al., 2007) in the T-DNA (Figure 2A). Twelve reps of three plates each, 16–20 explants per plate, were initiated. The total explant number ranged from 50 to 60 per rep. After co-cultivation, the explants were removed from the *Agrobacterium* suspension, placed onto medium 70A containing 5 mg/L spectinomycin for shoot initiation and selection. After 10–14 days on selection, the internodal segments formed a dumbbell shape with callus formation at each cut end of the segment. Green leafy shoots were observed after 4 weeks from the apical end of the internodal segments. Putative transgenic shoots were green while non-transgenic shoots on spectinomycin selection were bleached and white (Figure 3A). Wild-type shoots on media with no selection remained green and no evidence of bleaching (Figure 3B).

After two rounds of selection, 4 weeks in total, approximately 30% of the explants produced spectinomycin-resistant shoots, identified by a non-bleached phenotype and expression of TagRFP (Figures 3C,D). Regenerating wild-type shoots were green and had minimal autofluorescence (Figures 3E,F). Hyperhydricity of the shoots was commonly observed. Only ∼20–30% of the shoots exhibited a normal shoot phenotype with opaque, waxy leaves (Figure 3G). Up to 80% of the shoots exhibited some degree of hyperhydricity, as evidenced by leaves with a translucent, glassy appearance (Figure 3C, arrow). The developing shoots were transferred to shoot elongation medium 70C containing 10 mg/L spectinomycin for 2 weeks (Figure 3G) and placed back into the high light condition. Healthy, regenerating shoots 1 cm or longer were transferred to a rooting medium 90A without spectinomycin. Approximately 30–50% of the shoots produced roots and fully regenerated into plants. Green, transgenic rooted plants (Figure 3H) were collected and transferred to the greenhouse, and grown to maturity. T0 plants were sampled and analyzed by qPCR for the presence of the *spcN* and the TagRFP genes. Using the protocol as described above, T0 plants were successfully regenerated that expressed TagRFP (Figure 3D) and the selectable marker in the elite canola variety 4PYZE50B. The transformation frequency averaged 15.6%, with a range from 3.8 to 31.4%, and the escape rate was less than 10% (Table 5). The total duration of the transformation process was ∼66 days from the time of initial explant preparation and *Agrobacterium* infection until plants were suitable to be sent to the greenhouse for transplantation (see Supplementary Figure 2).

### Testing Hypocotyl and Epicotyl Explants From Multiple Genotypes

Preliminary screening of hypocotyl and epicotyl explants using the base protocol indicated 3–4 mm was the preferred explant size for optimal tissue culture response. Hypocotyl and epicotyl segments from germinated seedlings of multiple genotypes were evaluated to find the preferred explant that provided a reliable, high-frequency shoot formation response in tissue culture. Epicotyl segments consistently produced a statistically significant higher rate of shoot formation compared to hypocotyl segments across all genotypes tested (Figures 1B,C). Epicotyl segments even produced shoots from one genotype (NS7627MC) that was unable to generate shoots from hypocotyl explant (Figure 1C). The shoots from epicotyl segments emerged rapidly and were observed by week 4–5 after initiation, compared to 6–7 weeks or longer for shoots to appear from hypocotyl segments. Stem segments from the next higher internode were also evaluated for tissue culture response and they responded similarly to epicotyl segments (Supplementary Figure 1). For subsequent transformation experiments, epicotyl and stem segments were both utilized and are collectively called “internodal” segments. In a typical experiment, one seedling would produce 7–8 epicotyl explants and 1–2 explants were derived from the next higher internode.

To confirm the improved responsiveness of epicotyl explants compared to hypocotyl explants, two elite cultivars, female line 4PYZE50B and a male line, G00555MC, were chosen to test transformation efficiency with research vector RV008033 using spectinomycin selection (Table 2). Epicotyl segments consistently produced 5 to 10-fold higher T0 plant frequency than hypocotyl segments, 15.6 and 25.2 versus 2.9 and 1.9%, respectively.

**TABLE 2:** Comparison of transformation efficiency between epicotyl and hypocotyl explants using RV008033 in two elite canola cultivars.

Genotype	Construct	Explant	# Explant	# T0 plant	T0%
4PYZE50B(F)	RV008033	Epicotyl	629	98	15.6
4PYZE50B(F)	RV008033	Hypocotyl	577	17	2.9
G00555MC(M)	RV008033	Epicotyl	107	27	25.2
G00555MC(M)	RV008033	Hypocotyl	678	13	1.9

*%T0 = (#T0/#explant) × 100.*

### Impact of Explant Size, Temperature and Light Intensity on Internodal Segment Transformation

Using the base protocol, internodal segments from genotype 4PYZE50B were dissected into lengths of either 1, 3, 6, and 10 mm, transformed with RV008033 and scored for shoot response (Table 3). Internodal segments 3 mm long consistently produced the highest percentage of shoots per explant. Segment lengths between 3 and 4 mm were used for all subsequent experiments.

**TABLE 3:** Comparison of internodal explant size from 21 days old seedlings for shoot response using genotype 4PYZE50B.

Rep	Genotype	Size explant (mm)	# Explant	# Shoot	% Shoot?
1	4PYZE50B	1	89	16	18.0
	4PYZE50B	3	72	21	29.2
	4PYZE50B	6	25	1	4.0
	4PYZE50B	10	20	0	0.0
2	4PYZE50B	1	67	27	40.3
	4PYZE50B	3	24	17	70.8
	4PYZE50B	6	21	10	47.6
	4PYZE50B	10	20	0	0.0

*%Shoot = (#shoot/#explant) × 100.*

To evaluate the impact of light quality and intensity on explant responsiveness, canola seeds from genotype 4PYZE50B were germinated on half-strength MS medium, 90 base (Supplementary Table 1), at 26°C under different light intensities and light source (Table 4) for 28 days to obtain an epicotyl 3–4 cm in length. Explants and transformation were prepared and carried out as described above with three replications. Explants from seedlings grown under LED lights at an intensity of 60 μmol/m^2^/s produced a significantly higher percentage of shoots per explant (Table 4). Source seedlings for explants for all subsequent experiments were grown under LED lights.

**TABLE 4:** Light intensity and light quality impact on percentage of shoots per internodal explant from genotype 4PYZE50B, 21 days seedlings.

			Shoot %			
Genotype	Light	Light intensity (μmol/m^2^/s)	Rep 1 (%)	Rep 2 (%)	Rep 3 (%)	Average (%)
4PYZE50B	LED	60	80	58	53	64
4PYZE50B	Fluorescent	45	21	29	24	25
4PYZE50B	Dark	<1	0	0	0	0

*ANOVA single factor, P-value < 0.01; %shoot = (#shoot/#explant) × 100.*

### Transformation of Multiple Genotypes

To further test the effectiveness of the initial, base transformation protocol on different canola lines, internodal segments of seven genotypes, both male (M) and female (F) lines, were infected with the auxotrophic *Agrobacterium* strain LBA4404 THY-containing research vector RV008033. Transformation data for 4PYZE50B are described in Table 5 and other six genotypes in Table 6. All genotypes tested were transformable using this protocol, without further modification, at frequencies ranging from 3.5 to 33.3%. To further confirm the robustness of the protocol, 12 genotypes representing a range of breeding germplasm, both male (M) and female (F) lines, were tested for transformation efficiency using the optimized protocol for internodal segments (seedlings grown under LED light at 60 μmol/m^2^/s, 3–4 mm explants and *spcN* selection). A vector designed for commercial transformation purposes, RV028667 (Figure 2B) was used for this experiment. All 12 genotypes were successfully transformed with efficiencies ranging from 1.0 to 33.3% and were confirmed as having an intact T-DNA and copy number was determined by qPCR for DsRed and the *spcN* selectable marker (Table 7). Several of the genotypes, 4PYWY36B, 4PQRA43B, G00555MC, 4PBUG16R produced shoots that exhibited less hyperhydricity and had shoots with better, more well-organized meristems, as evidenced by the presence of obvious young leaf primordia (Figure 3C). Shoots that were well-organized readily regenerated into plants even if they exhibited some hyperhydricity.

**TABLE 5:** Transformation frequencies from internodal explants obtained in canola genotype 4PYZE50B, 21 days seedlings transformed with RV008033.

Replicate	# Explant	# T0 (% T0)	# PCR+ (% PCR+)
1	52	3(5.8)	89 (90.8)
2	52	2(3.8)	
3	53	7(13.2)	
4	54	10(18.5)	
5	60	11(18.3)	
6	52	9(17.3)	
7	51	16(31.4)	
8	50	8(16.0)	
9	50	7(14.0)	
10	52	10(19.2)	
11	52	6(11.5)	
12	51	9(17.6)	

*qPCR was performed on T0 plants in the greenhouse to detect the presence of the selectable marker (spcN), the left (PSB1), and right border (PSA2) regions, and the absence of Agrobacterium tumefaciens backbone sequences. %T0 = (#T0/#explant) × 100; %qPCR + = (#qPCR/#T0) × 100.*

**TABLE 6:** Multiple genotype test using RV008033 and internodal explants from 21 days seedlings.

Genotype	# Explant	# T0	T0 %
4PUCC04B(F)	165	55	33.3
G00555MC(M)	107	27	25.2
NS1822BC(F)	204	11	5.4
4PUSL90B(F)	285	27	9.5
4PQRA43B(F)	30	2	6.7
Westar	85	3	3.5

*F, female genotype; M, male genotype.*

**TABLE 7:** Transformation frequencies from internodal explants in 12 elite canola genotypes using RV028667 and 25 days seedlings for explants.

RV #	Genotype	# Explant	# T0 (%T0)	# QE (%QE)
RV028667	4PYZE50B(F)	113	8(7.1)	6(75)
RV028667	NS1822BC(F)	100	6(6.0)	3(50)
RV028667	4PYWY36B(F)	100	4(4.0)	1(25)
RV028667	NS7627MC(M)	52	1(1.9)	1(100)
RV028667	4PQRA43B(F)	91	2(2.2)	0(0)
RV028667	NS8245MC(M)	100	1(1.0)	0(0)
RV028667	4PUCC04B(F)	97	22(22.7)	6(27)
RV028667	G00555MC(M)	31	6(19.4)	3(50)
RV028667	4PBUG16R(M)	128	5(3.9)	3(60)
RV028667	4PXJR06B(F)	67	15(22.4)	9(60)
RV028667	4PUSL90B(F)	100	14(14.0)	3(21)
RV028667	G00182MC(M)	9	3(33.3)	2(67)

*F, female line; M, male line; QE, single copy quality event without any vector backbone. %QE = (#QE/#T0) × 100.*

### Gene Editing Using CRISPR-Cas9

To demonstrate the applicability of the protocol for genome editing, internodal segments from canola genotype 4PYZE50B were infected with the auxotrophic *Agrobacterium* strain LBA4404 THY- harboring plasmid RV029164 targeting the canola *COMT* gene (Figure 2C). Spectinomycin selection was used to identify transgenic events at a 4% frequency. To confirm editing, 10 T0 plants were evaluated for site-specific mutations using NGS. As shown in Table 8, a mutation frequency of 100% for the *COMT* target sites of both the A and C genome was observed in plant 3. Robust mutation frequencies of 50–80% were also observed in plant 6 and 7, while 8–24% mutation rates were observed in four plants (plant 2, 4, 9, and 10). No mutations for either the A or C genome were observed in three plants (plant 1, 5, and 8). Majority of the mutations were 1–4 nucleotide deletions at the predicted cut site, a single nucleotide insertion was also observed in alleles of line 6 and 7 (Supplementary Table 4).

**TABLE 8:** Mutation frequency at COMT target site in the A and C genome.

T0 plant	NGS reads		A genome reads			C genome reads		
	Total	Filtered	Total	Mutation	% Mutation	Total	Mutation	% Mutation
1	36,695	29,426	18,852	0	0	10,574	0	0
2	54,159	40,991	19,654	2268	12	21,337	1795	8
3	45,013	36,758	12,058	12,058	100	24,700	24,700	100
4	41,887	30,936	14,635	3959	27	16,301	3958	24
5	52,860	41,895	20,734	0	0	21,161	0	0
6	41,328	30,672	15,424	12,370	80	15,248	10,876	71
7	34,380	23,215	11,009	5714	52	12,206	5599	46
8	41,152	33,429	16,313	0	0	17,116	0	0
9	33,749	25,222	12,188	1740	14	13,034	1519	12
10	41,221	30,724	14,598	1902	13	16,126	1969	12

### T0 Fertility and T1 Segregation Analysis

The transgene was successfully transmitted to the T1 generation in all the transgenic events analyzed to date (several hundred total). T1 plants produced seed >99% of the time (2 sterile plants out of 1033, to date) with seed set ranging from 500 to 3500 seed per plant. Average seed set was ∼2000 seed per plant. Transgenic T0 events generated early in the development of the protocol were analyzed by qPCR with only “presence/absence” data to indicate transgene integration. T0 events sent to the greenhouse could potentially be single-copy, multi-copy, or chimeric. T1 segregation data identified three clear classes of segregation based on Chi-square analysis (Supplementary Table 5). One class of plants was consistent with a single copy integration event and normal Mendelian 3:1 inheritance. Class 2 deviated from the expected 3:1 segregation by having an excess number of null plants. This suggests that some T1 plants were chimeric, a common observation with plants regenerated via organogenesis. A third class of events had segregation ratios that were consistent with two independent integration events.

## Discussion

The goal of this study was to develop a transformation and genome editing technology that was simple to execute, robust, efficient across multiple elite genotypes and scalable for reasonably high-throughput production transformation of commercially relevant parental lines used to produce hybrid canola. We chose to use aseptically germinated seedlings as the source of explant material as they are readily obtained, can be grown as a continuous supply and provide a variety of explants to test. As our base protocol, we reproduced, with a few modified steps, the De Block et al. (1989) method with hypocotyl segments and then tested other parts of the germinated seedling as potential transformation targets, including epicotyl segments, higher internode segments, petioles and leaves. We also tested the *spcN* gene that confers resistance to the antibiotic spectinomycin as a selectable marker for canola.

In our initial experiments looking at the tissue culture response from explants derived from a germinated seedling, we identified tissue segments from canola epicotyls and higher internodes to be particularly responsive to tissue culture and *in vitro* shoot formation. Compared to hypocotyl explants, internodal explants responded more quickly to tissue culture, producing visible shoots within 4–5 weeks, compared to 6–7 weeks for hypocotyl explants. Shoot formation frequency was also typically much higher from internodal segments, ranging from 10 to 30% while hypocotyl segments were typically less than 5% responsive. The quality of the seedling used to generate the explants was important for a robust tissue culture response. The quality and intensity of light was found to be particularly important with LED lights with a light spectrum of approximately 20% blue, 40% green, 40% red at an intensity of 60 μmol/m^2^/s or higher providing a sufficient quantity of internodal explants with consistent responsiveness.

Similarly, the size of the explant was determined to be important for optimal responsiveness from internodal segments. In many of the protocols that utilize hypocotyls (De Block et al., 1989; Cardoza and Stewart, 2003; Maheshwari et al., 2011), the explant size is typically 10 mm. We found the optimal explant size for internodal segments to be 3–4 mm, which meant that we could produce three times as many explants from a given length of internode when compared to hypocotyls. This was important as both the epicotyl and higher internodes in the germinated seedlings were considerably shorter than the hypocotyls. By using shorter explant segments, this allowed for a much greater number of explants per seedling, typically 8–10 per seedling, of which 7–8 were epicotyl segments, supplemented with 1–2 explants derived from higher internodes.

Shoots consistently formed from the apical end of the explants with more callus formation appearing at the basal end. Presumably, this reflects continued endogenous basal auxin transport in the epicotyl segments with auxin accumulating at the basal end, leading to more callus formation and a reduced auxin environment at the apical end, thereby enabling shoot formation. Consistent with other published canola transformation protocols, hyperhydricity and poor-quality shoot formation was an issue with this protocol as well. While efforts continue to lessen the impact of hyperhydricity, the current protocol is robust and efficient enough to allow for the efficient production of transgenic shoots across all genotype tested.

Transgenes and genome edits were successfully transmitted to the T1 generation with 100% efficiency. The segregation data of the early generated events suggests that events with single-copy, multi-copy and possible chimeric plants were regenerated. This has been obviated in later generation events derived from the commercial vector where copy-number analysis was done at the T0 generation. When single-copy, no vector backbone events were sent to the greenhouse for T1 seed production, T1 segregation data was consistent with single gene integration and Mendelian segregation.

The hypocotyl system, while efficient for certain genotypes such as Westar, has been difficult to apply to elite, commercial germplasm. In one example, modifications to the hypocotyl system were described by Maheshwari et al. (2011) where they were able to extend the protocol to two genotypes, Topas and Line 4079, that had previously been unable to transform using Westar-like methods. They achieved this by employing a scorable marker, luciferase, and not utilizing a selectable marker to identify transgenic events. They also calculated their transformation efficiency based on the number of transgenic events from the total number of events regenerated, with efficiencies ranging from 54.2% for Invigor to 13.4% for Line 4079. This means that from ∼85–50% of the plants produced using the luciferase marker were escapes. While their protocol was an improvement over previous hypocotyl-based methods, the need to screen for a scorable marker and the high escape rate make this method impractical for high-throughput, production transformation purposes.

Using the protocol that had been optimized for transformation, we further tested the protocol for its application to CRISPR-Cas genome editing using the *COMT* genomic site for creating targeted mutagenesis. Among 10 T0 plants regenerated (Table 7), mutations at *COMT* sites of both genomes were detected in seven plants (70%) by NGS. Biallelic mutations in both genomes were observed in two plants. The mutation frequencies in both canola genomes observed in a small number of T0 regenerated plants in this study appear to be higher than previously reported in *B. napus* (Zhai et al., 2019). Among more than 200 T0 plants regenerated in this previous report, positive mutations at target sites were observed in a small number of plants. In a different genome editing study in *B. napus*, only 2 out of 22 T0 plants analyzed were detected positive for targeted mutations at *fatty acid desaturase 2* gene (Okuzaki et al., 2018). Similarly, mutation in 20–56% of T0 plants was reported in *Brassica campestris* (Xiong et al., 2019). However, Yang et al. (2017) have reported 65.3% mutation frequency in the T0 generation, similar to the frequencies observed in this study.

The optimized transformation and genome editing method described here, utilizing 3–4 mm internodal segments from seedling grown under LED light and *spcN* selection, produced transgenic or edited events in every genotype tested with very few escapes, typically less than 5%. The process is simple, robust, and rapid and it produced healthy, fertile, transgenic or edited plants that exhibited normal Mendelian segregation in the T1 generation. The protocol has been successfully deployed in Corteva’s commercial production lab and has, over the past 2 years, produced over a 1000 events, in both male and female canola lines.

Hyperhydricity, a common problem with canola tissue culture, and shoots with poorly organized meristems was still commonly observed, however. Addressing that shortcoming remains an active area of research, as does increasing the overall efficiency. Still, the protocol described here has proven to be a very robust and efficacious method for the production of transgenic and genome edited events in multiple genotypes representing both male and female parents of commercial canola hybrids.

## Data Availability Statement

Materials reported in this article may contain components subject to third party ownership (e.g., TagRFP and Ds-RED). Transgenic and genome edited materials may be subject to governmental regulations. Availability of materials described in this article to academic investigators for non-commercial research purposes under an applicable material transfer agreement will be subject to proof of permission from any third-party owners of all or parts of the material and to governmental regulation considerations. Obtaining the applicable permission from such third-party owners will be the responsibility of the requestor. Transgenic materials reported in this article may only be made available if in full accordance with all applicable governmental regulations.

## Author Contributions

TJ, SK, and UC conceived and designed the experiments, supervised the execution of research, and wrote the manuscript. UC, KJ, YL, and PV conducted transformation experiments. AS contributed to genome editing experiments. All authors critically reviewed and approved the manuscript.

## Conflict of Interest

The authors are Corteva Agriscience employees.
