# Supplementary figures and images for: Genotype-Independent Transformation and Genome Editing of Brassica napus Using a Novel Explant Material
Source: Front Plant Sci. 2020 Oct 8;11:579524. doi: 10.3389/fpls.2020.579524 (PMC7578431; doi:10.3389/fpls.2020.579524)

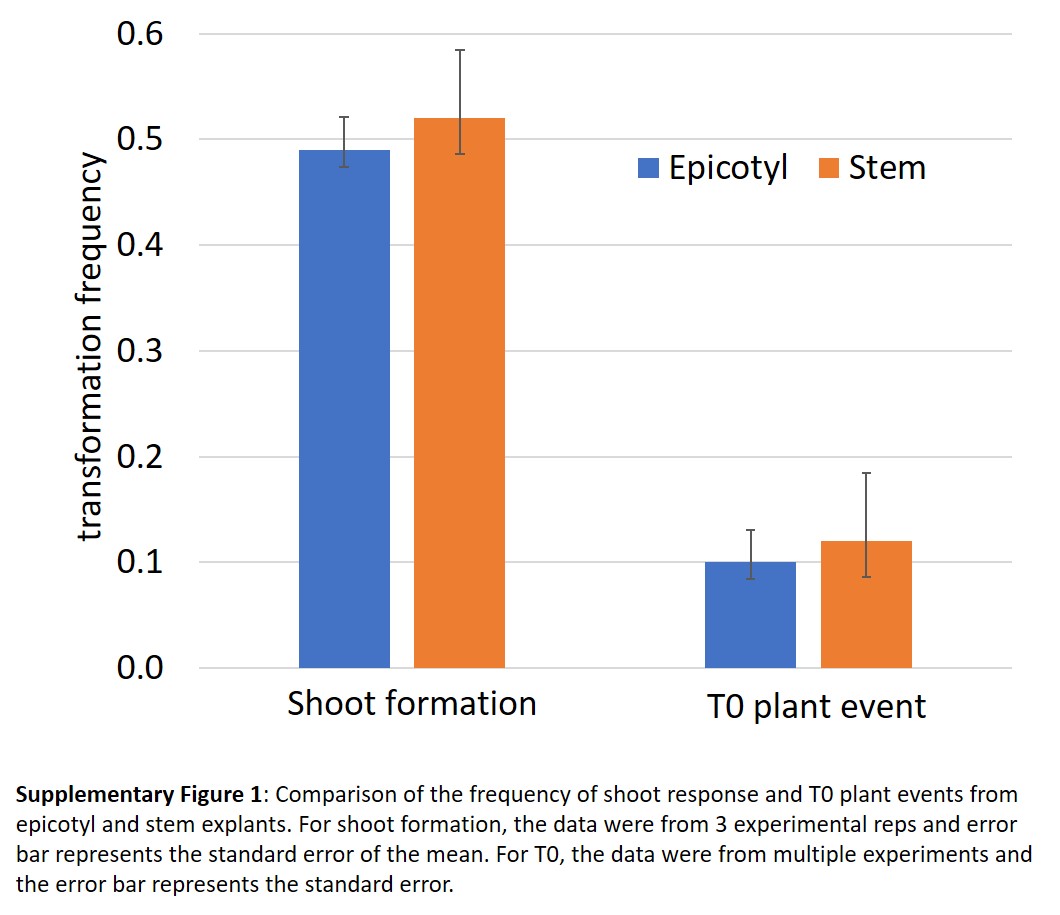

Supplement: Supplementary file 1 [file Image_1.jpg]

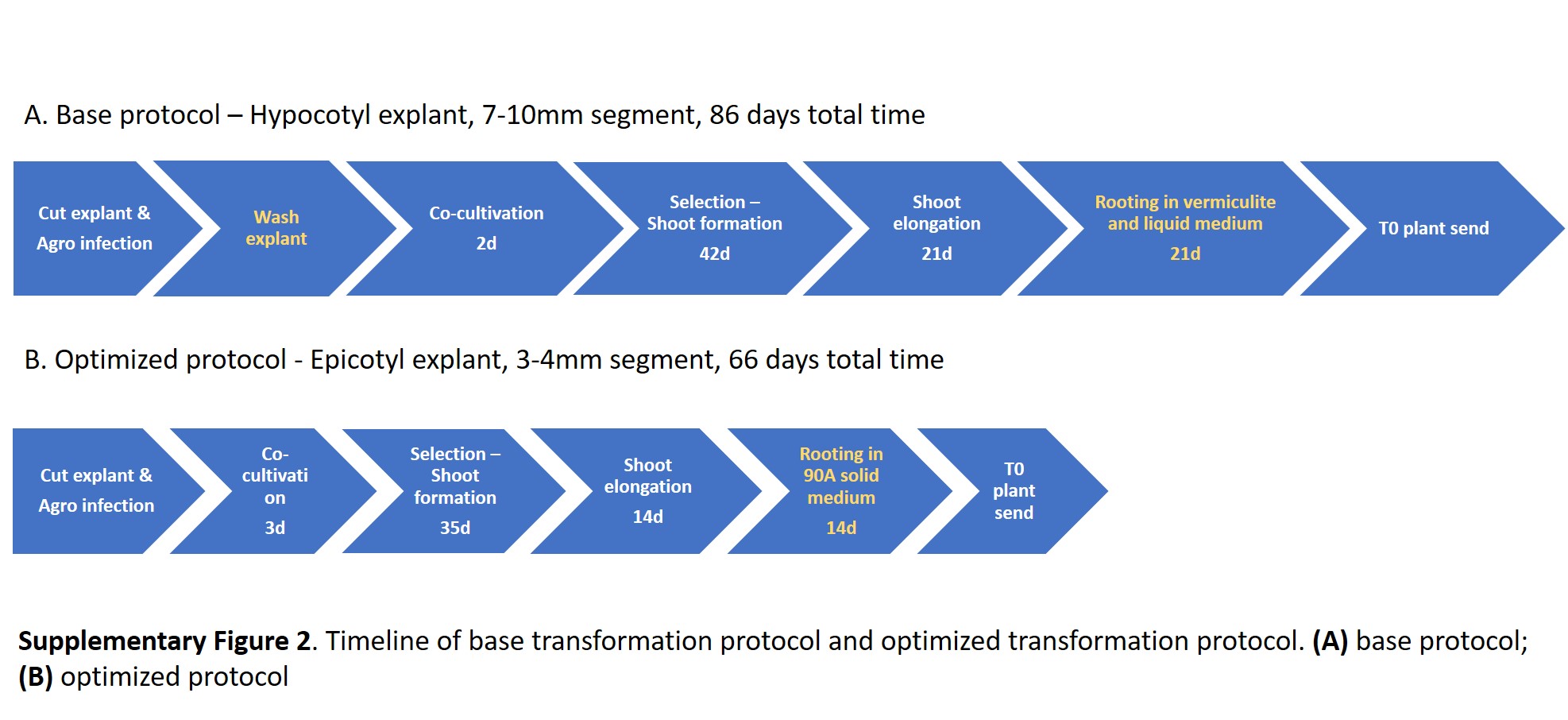

Supplement: Supplementary file 2 [file Image_2.jpg]
